# A nomogram for predicting the rapid progression of diffuse large B‐cell lymphoma established by combining baseline PET/CT total metabolic tumor volume, lesion diffusion, and *TP53* mutations

**DOI:** 10.1002/cam4.6295

**Published:** 2023-06-27

**Authors:** Cong Liu, Pengyue Shi, Zhenjiang Li, Baosheng Li, Zengjun Li

**Affiliations:** ^1^ National Clinical Research Center for Cancer, Key Laboratory of Cancer Prevention and Therapy, Tianjin, Tianjin's Clinical Research Center for Cancer Tianjin Medical University Cancer Institute and Hospital Tianjin China; ^2^ Department of Radiation Oncology Tianjin Medical University Tianjin China; ^3^ Department of Internal Medicine‐Oncology, Shandong Cancer Hospital and Institute Shandong First Medical University and Shandong Academy of Medical Sciences Jinan Shandong China; ^4^ Department of Radiation Oncology, Shandong Cancer Hospital and Institute Shandong First Medical University and Shandong Academy of Medical Sciences Jinan Shandong China; ^5^ Department of Radiation Oncology Physics and Technology, Shandong Cancer Hospital and Institute Shandong First Medical University and Shandong Academy of Medical Sciences Jinan Shandong China; ^6^ Department of Hematology, Shandong Cancer Hospital and Institute Shandong First Medical University and Shandong Academy of Medical Sciences Jinan Shandong China

**Keywords:** diffuse large B‐cell lymphoma, nomogram, positron emission tomography/computed tomography, *TP53*

## Abstract

**Objectives:**

This study aimed to integrate positron emission tomography/computed tomography (PET/CT) metrics and genetic mutations to optimize the risk stratification for diffuse large B‐cell lymphoma (DLBCL) patients.

**Methods:**

The data of 94 primary DLBCL patients with baseline PET/CT examination completed in the Shandong Cancer Hospital and Institute (Jinan, China) were analyzed to establish a training cohort. An independent cohort of 45 DLBCL patients with baseline PET/CT examination from other hospitals was established for external validation. The baseline total metabolic tumor volume (TMTV) and the largest distance between two lesions (Dmax) standardized by patient body surface area (SDmax) were calculated. The pretreatment pathological tissues of all patients were sequenced by a lymphopanel including 43 genes.

**Results:**

The optimal TMTV cutoff was 285.3 cm^3^ and the optimal SDmax cutoff was 0.135 m^−1^. *TP53* status was found as an independent predictive factor significantly affecting complete remission (*p* = 0.001). TMTV, SDmax, and *TP53* status were the main factors of the nomogram and could stratify the patients into four distinct subgroups based on their predicted progression‐free survival (PFS). The calibration curve demonstrated satisfactory agreement between the predicted and actual 1‐year PFS of the patients. The receiver operating characteristic curves showed this nomogram based on PET/CT metrics and *TP53* mutations had a better predictive ability than the clinic risk scores. Similar results were identified upon external validation.

**Conclusions:**

The nomogram based on imaging factors and *TP53* mutations could lead to a more accurate selection of DLBCL patients with rapid progression, to increase tailor therapy.

## Graphical abstract

In the era of immunity and targeted therapy, the existing International Prognostic Index scoring system has failed to fully identify high‐risk population for diffuse large B‐cell lymphoma (DLBCL). By analyzing the positron emission tomography/computed tomography parameters and next‐generation sequencing of primary DLBCL patients in the training cohort and validation cohort, we found the nomogram based on imaging factors and *TP53* status could lead to a more accurate selection of DLBCL patients with rapid progression, to increase tailor therapy.

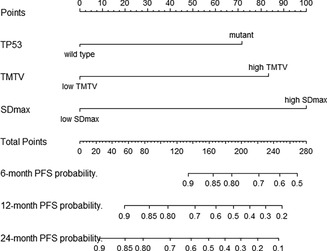

## 1 INTRODUCTION

Diffuse large B‐cell lymphoma (DLBCL) is the most prevalent type of non‐Hodgkin's lymphoma, accounting for 40% of all lymphomas. It is a highly heterogeneous disease entity with differing prognoses.^1^ Almost 40% of patients experience relapse/metastasis following first‐line standard treatment, and survival is particularly poor for patients relapsing within 1 year after R‐CHOP, with <15% achieving durable remission.^2^, ^3^ Although the International Prognostic Index (IPI), age‐adjusted‐IPI (aa‐IPI), and National Comprehensive Cancer Network IPI (NCCN‐IPI) models remain the basis for prognostic evaluation and treatment stratification in DLBCL, several studies have reported that these clinical scores failed to fully identify extremely high‐risk populations in the rituximab era,^4^, ^5^, ^6^ resulting in these patients missing the opportunity to receive new treatment regimens at an early stage to effectively prolong the survival.^7^, ^8^, ^9^ Therefore, new prognostic models are needed as a benchmark for determining the prognosis and guiding novel treatment regimens for DLBCL patients.

Fluorodeoxyglucose (^18^F)‐positron emission tomography/computed tomography (PET/CT) is currently recognized as the most accurate imaging tool for staging and evaluating the treatment response of DLBCL. Baseline total metabolic tumor volume (TMTV) is a good indicator of prognosis, reflecting the baseline tumor burden and metabolism.^10^, ^11^ A high baseline TMTV results in significantly shorter progression‐free survival (PFS) and overall survival (OS) in many lymphoma subtypes, including DLBCL.^12^, ^13^ In addition, the largest distance between two lesions (Dmax) calculated and normalized by patient body surface area (SDmax), as a simple imaging feature measured on PET scans, is a prognostic factor independent of TMTV that reflects lesion dissemination.^14^ However, it is not comprehensive to use imaging indexes solely to predict the curative effect without considering molecular heterogeneity.

Recently, major breakthroughs have been made in research on gene expression profiles of DLBCL with therapeutic implications.^15^, ^16^, ^17^ The emergence of next‐generation sequencing (NGS) over the past decade has enabled high‐through put DNA sequencing, and the heterogeneity of DLBCL has been analyzed based on genetic alterations.^18^ Wright et al.,^16^ divided DLBCL into seven genomic subtypes to analyze the heterogeneity of DLBCL and aid the development of rationally targeted therapy. Multiple gene mutations, especially *TP53* mutations, are important in guiding the selection and efficacy of drugs and are closely related to prognosis.^19^ Even so, genetic molecules have not been included in the current clinical risk scoring system, which may result in many genetic high‐risk patients missing the opportunity to receive adequate treatment at an early stage.

No previous study had explored an integrated prognostic model that combines imaging and genetic molecular factors. This study aimed to establish and validate the nomogram based on PET/CT metrics and genetic mutations for optimizing the prediction of high‐risk DLBCL population.

## 2 METHODS

### 2.1 Study population

We retrospectively collected the clinical data of 152 primary DLBCL adult patients (age ≥18 years) diagnosed between April 2019 and February 2022 in the Shandong Cancer Hospital and Institute (Jinan, China). The main endpoints were the complete remission (CR) rate and PFS after first‐line chemotherapy. The inclusion criteria were as follows: (1) DLBCL confirmed in all patients by histopathological review of the baseline biopsy; (2) the pretreatment pathological tissues of all patients were sequenced by a lymphopanel including 43 genes; (3) the data of baseline ^18^F‐PET/CT inspection available; (4) All patients were treated by R‐CHOP (rituximab, cyclophosphamide, doxorubicin, vincristine, and prednisone) or R‐CHOP‐like chemotherapy, including 28 patients diagnosed with double expressor DLBCL who came from a prospective, single‐arm, phase II clinical trial, and received zanubrutinib combined with R‐CHOP regimen. The initial results of the prospective study had been presented by ASH in 2022. The main exclusion criteria were incomplete systemic chemotherapy for at least 4 cycles or only one lesion on PET/CT. The patients were followed up monthly by review of hospital electronic medical records and telephone calls. The last follow‐up period was up to December 2022. Finally, 139 patients were included in the discussion and analysis. Among them, 94 patients with baseline PET/CT examination completed in the Shandong Cancer Hospital and Institute were allocated to establish a training cohort. An independent cohort of 45 DLBCL patients with baseline PET/CT examination from other hospitals was established for external validation.

This study was approved by the Medical Ethical Committee of Shandong Cancer Hospital and Institute (No. SDTHEC2022007008). Written informed consent for participation was not required for this retrospective study in accordance with the national legislation and the institutional requirements. The waiver of informed consent was approved by the Medical Ethical Committee of Shandong Cancer Hospital and Institute. The study was conducted in accordance with the Declaration of Helsinki (as revised in 2013).

### 2.2 Variables and definitions

The clinical data obtained from all patients included: sex, age at disease onset, Eastern Cooperative Oncology Group (ECOG)‐point scale (PS), Ann Arbor stage at diagnosis, bulky disease, IPI, aa‐IPI, and NCCN‐IPI at diagnosis, histological classification, BCL2/MYC double expression, lactate dehydrogenase (LDH) level, initial chemotherapy, treatment response, pretreatment PET/CT images, and 43 gene mutations based on NGS. PFS was calculated from the date of randomization to the date of death from any cause, disease relapse or progression, or the date of last follow‐up. CR was defined based on PET/CT treatment response to at least four cycles of R‐CHOP‐like chemotherapy according to the Lugano criteria, as follows: Score 1, 2, or 3 with or without a residual mass on 5‐PS (1, no uptake above background; 2, uptake ≤ mediastinum; 3, uptake > mediastinum but ≤ liver; 4, uptake moderately > liver; 5, uptake markedly higher than liver and/or no new lesions; X, new areas of uptake unlikely to be related to lymphoma), no new lesions and no evidence of FDG‐avid disease in marrow.

### 2.3 Baseline PET metrics

TMTV was defined by two nuclear medicine physicians (blinded to patient outcomes) using a 41% maximum standardized uptake value (SUVmax) threshold on artificial intelligence‐assisted medical image auto‐delineation (AccuContour version 3.2; ManteiaTech). Bone marrow involvement was included in the volume measurement only if there was focal uptake. The spleen was deemed to be involved and included if there was focal uptake or diffuse uptake >150% of the liver background. From the 3‐dimensional (3D) coordinates of the metabolic volume of each lymphoma lesion, the center of mass (centroid) of each lesion was automatically obtained using AccuContour and was taken as the lesion location. The distances between all pairs of lesions were calculated using the Euclidian formula.

(1) $$\mathrm{AB}=\sqrt{{\left(\mathrm{xb}-\mathrm{xa}\right)}^{2}+{\left(\mathrm{yb}-\mathrm{ya}\right)}^{2}+{\left(\mathrm{zb}-\mathrm{za}\right)}^{2}}$$

Dmax was calculated in each patient and normalized by the patient's body surface area.

(2) $$\sqrt{\left(\text{weight}\times \text{height}\right)/3600}$$

### 2.4 NGS data analysis

Based on a literature review of the NGS studies on DLBCL,^20^, ^21^, ^22^, ^23^ a panel was designed which included 43 genes: *ARID1B*, *ATM*, *B2M*, *BCL10*, *BCL2*, *BCL6*, *BTG1*, *CARD11*, *CCND3*, *CD58*, *CD79A*, *CD79B*, *CDKN2A*, *CIITA*, *CREBBP*, *EPHA7*, *EZH2*, *EP300*, *FAS*, *GNA13*, *IRF8*, *ITPKB*, *JAK2*, *KMT2C*, *KMT2D*, *MEF2B*, *MFHAS1*, *MYC*, *MYD88*, *NOTCH1*, *NOTCH2*, *PAX5*, *PIM1*, *PPM1D*, *SOCS1*, *SGK1*, *STAT3*, *STAT6*, *TET2*, *TNFAIP3*, *TNFRSF14*, *TP53*, and *XPO1*. The lymphopanel was designed by KingMed Diagnostics. Genomic DNA was extracted from formalin‐fixed, paraffin‐embedded tumor tissue from patients with DLBCL using a QIAamp DNA FFPE Tissue Kit (Qiagen). Additionally, polymerase chain reaction (PCR) primers were designed by IDT, and high‐throughput DNA sequencing was performed on the Illumina Novaseq 6000.

### 2.5 Statistical analysis

The threshold to determine the TMTV and SDmax optimal cutoff values of the quantitative parameters for PFS prediction was tested by the receiver operating characteristic (ROC) curve analysis. The baseline characteristics of the 2 groups were analyzed using Pearson's χ^2^ or Fisher's exact tests. Survival functions were estimated using the Kaplan–Meier (KM) method and compared by log‐rank test. The median follow‐up was estimated by the reverse KM method. Univariate and multivariate analyses were performed using Cox proportional hazards models and logistic regression models. In the training cohort, risk factors selected for univariate analyses were based on previous studies and were routinely available in clinical practice. Considering the limited number of patients by genotyping and significance in univariate analysis, variables selected for multivariable Cox regression included Ann Arbor stage, TMTV, SDmax, aa‐IPI, and *TP53* status. According to the results of multivariate Cox regression analysis, a nomogram prediction model of PFS was established. Calibration curves were derived based on regression analyses to determine whether the predicted probability was consistent with the actual survival of the patients. Comparisons of the predictive ability between the nomogram with IPI, aa‐IPI, and NCCN‐IPI were investigated by the area under the ROC curves (AUC).

Statistical analysis was performed using the survival, survminer, ggplot2, ROC, and rmda packages in R, version 4.2.2 (http://www.r‐project.org/). All *p*‐values were 2‐sided and those less than 0.05 were considered statistically significant.

## 3 RESULTS

### 3.1 Patient characteristics

The baseline characteristics of DLBCL patients in each cohort are listed in Table 1. The median follow‐up duration of the training and validation cohort were 25.5 and 24.6 months, respectively. The TMTV of the 139 patients had a non‐normal distribution and the median TMTV was 249.0 cm^3^ (P25–P75, 121.2–610.6 cm^3^). ROC curve analysis showed that the optimal TMTV cutoff was 285.2 cm^3^ (Figure S1). The median Dmax was 0.31 m (P25–P75, 0.10–0.54 m), and SDmax was 0.150 m^−1^ (P25–P75, 0.060–0.250 m^−1^). ROC curve analysis showed that the optimal SDmax cutoff was 0.135 m^−1^ (Figure S1). The TMTV and SDmax were converted into binary variables according to the cutoff values. Patients in each cohort were divided into seven subtypes according to the NGS results based on Wright's study^16^: MCD‐like subtype (*MYD88* ^*L265P*^ and *CD79B* mutations); BN2‐like subtype (*NOTCH2* mutations or *BCL6* fusion); EZB‐like subtype (*EZH2* mutations or *BCL2* translocation); N1‐like subtype (*NOTCH1* mutations); A53‐like subtype (biallelic *TP53* mutations); ST2‐like subtype (*SGK1* and *TET2* mutations); others subtype. No correlation was observed between two cohorts of the seven subtypes (Table 1). The top five genes with the highest mutation rates were *PIM1*, *TP53*, *MYD88*, *CD79B*, and *KMT2D*, with their mutation rates 36.69%, 33.81%, 33.09%, 25.18%, and 25.18%, respectively, in the total population. The 43 gene mutation frequencies in the training cohort and in the validation cohort are presented in Table S1, and no significant differences in the 43 gene mutation frequencies were observed between two cohorts.

**TABLE 1:** Patients' clinical characteristics according to the training cohort and validation cohort.

Characteristic	Training cohort (*n* = 94)	Validation cohort (*n* = 45)	*p*‐Value
Female sex	43 (45.7%)	18 (40.0%)	0.523
Age >60 years	34 (36.2%)	12 (26.7%)	0.265
ECOG ≥2	13 (13.8)	9 (20.0)	0.351
Ann Arbor stage III–IV	65 (69.1%)	33 (73.3%)	0.613
Bulky disease (>10 cm)	20 (21.3)	8 (17.8)	0.632
aa‐IPI 2–3	59 (60.8)	27 (64.3)	0.700
Non‐GCB (Hans)^a^	36 (39.1%)	13 (30.2%)	0.316
Double expressor^b^	30 (32.6)	13 (28.9)	0.659
LDH >1 N	64 (68.1)	30 (66.7)	0.867
TMTV>285cm^3^	43 (45.7%)	21 (46.7)	0.919
SDmax >0.135 m^−1^	59 (62.8)	23 (51.1)	0.191
Complete remission	68 (72.3)	34 (75.6)	0.688
Genogroups			
MCD‐like	15 (16.0)	3 (6.7)	0.127
A53‐like	10 (10.6)	3 (6.7)	0.356
N1‐like	3 (3.2)	1 (2.2)	0.711
BN2‐like	4 (4.3)	2 (4.4)	0.959
EZH2‐like	2 (2.1)	1 (2.2)	0.971
ST2‐like	5 (5.3)	2 (4.4)	0.868
Others	55 (58.5)	33 (73.3)	0.09

Abbreviations: A53, biallelic *TP53* mutations; BN2, *BCL6* translocations and *NOTCH2* mutations; ECOG, Eastern Cooperative Oncology Group; EZH2, *EZH2* mutations and *BCL2* translocations; GCB, germinal center B; LDH, lactate dehydrogenase; MCD, *MYD88* and *CD79B*‐mutated; N1, *NOTCH1* mutations; R‐CHOP, rituximab combined with cyclophosphamide, doxorubicin, vincristine, and prednisone; ST2, *SGK1*, and *TET2* mutated; TMTV, total metabolic tumor volume.

^a^ BCL2/MYC double expression data available for 92 patients in the training cohort.

^b^ Histological classification data available for 92 patients in the training cohort and 43 patients in the validation cohort.

### 3.2 Independent prognostic factors affecting CR and PFS in the training cohort

The results of univariate and multivariate analysis for CR in the training cohort are listed in Table 2. At multivariate level, *TP53* status was found as an independent predictive factor significantly affecting CR (*p* = 0.001); TMTV on baseline PET/CT (hazard ratio [HR] 3.135, 95% CI: 1.495–6.576, *p* = 0.002), SDmax (HR: 4.467, 95% CI: 1.458–13.685, *p* = 0.009), and *TP53* mutations (HR: 2.854, 95% CI: 1.502–5.423, *p* = 0.001) were markedly associated with PFS (Table 3). No significant difference for CR and PFS was observed based on the other gene mutations or subtypes (Table S2).

**TABLE 2:** Univariate and multivariate analysis for CR in the training cohort.

Variable	Univariable logistic regression			Multivariable logistic regression		
	Odds ratio	95% Confidence interval	*p*‐Value	Odds ratio	95% Confidence interval	*p*‐Value
TMTV	4.976	1.832–13.515	0.002	3.090	0.926–10.318	0.067
SDmax	3.316	1.119–9.827	0.031	1.260	0.303–5.245	0.751
Ann Arbor stage	4.746	1.295–17.393	0.019	1.354	0.178–10.332	0.770
Double expressor	1.134	0.434–2.962	0.797	—	—	—
*TP53* ^*mut*^	6.750	2.490–18.301	<0.001	6.018	2.058–17.593	0.001
A53‐like	3.780	1.042–13.719	0.043	—	—	—
*MYD88* ^*L265P*^	0.356	0.110–1.155	0.085	—	—	—
MCD‐like	0.353	0.074–1.685	0.191	—	—	—
aa‐IPI	6.053	1.658–22.095	0.006	2.741	0.416–18.034	0.294

Abbreviations: aa‐IPI, age‐adjusted‐IPI; A53, biallelic *TP53* mutations; CR, complete remission; MCD, *MYD88/CD79B*‐mutated; NCCN‐IPI, National Comprehensive Cancer Network IPI; PFS, progression‐free survival; SDmax, standardized Dmax; TMTV, total metabolic tumor volume.

**TABLE 3:** Univariate and multivariate analysis for PFS in the training cohort.

Variable	Univariable Cox regression			Multivariable Cox regression		
	Hazard ratio	95% Confidence interval	*p*‐Value	Hazard ratio	95% Confidence interval	*p*‐Value
TMTV	4.918	2.450–9.875	<0.001	3.135	1.495–6.576	0.002
SDmax	5.913	2.314–15.108	<0.001	4.467	1.458–13.685	0.009
Ann Arbor stage	3.268	1.373–7.779	0.007	0.507	0.137–1.875	0.309
Double expressor	1.243	0.621–2.488	0.540	—	—	—
*TP53* ^*mut*^	2.590	1.392–4.818	0.003	2.854	1.502–5.423	0.001
A53‐like	2.432	1.071–5.527	0.034	—	—	—
*MYD88* ^*L265P*^	1.990	0.918–4.313	0.081	—	—	—
MCD‐like	2.008	0.715–5.643	0.186	—	—	—
aa‐IPI	3.320	1.470–7.497	0.004	2.146	0.705–6.528	0.179

Abbreviations: aa‐IPI, age‐adjusted‐IPI; PFS, progression‐free survival; SDmax, standardized Dmax; TMTV, total metabolic tumor volume.

### 3.3 Establishment and validation of the nomogram

**FIGURE 1:**
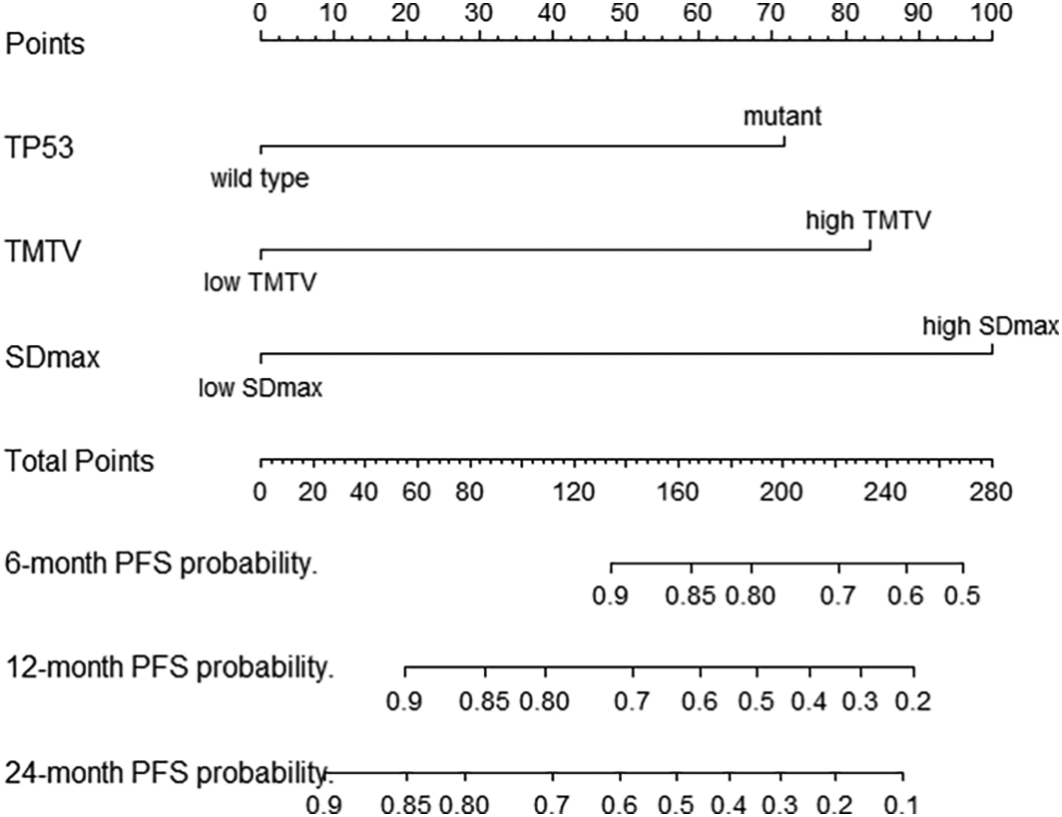
Nomogram established from the training cohort for predicting PFS of DLBCL patients by combining baseline TMTV, SDmax, and *TP53* mutations. DLBCL, diffuse large B‐cell lymphoma; Dmax, the largest distance between 2 lesions; high SDmax, SDmax >0.135 m^−1^; high TMTV, TMTV>285cm^3^; low SDmax, SDmax ≤0.135 m^−1^; low TMTV, TMTV ≤285cm^3^; SDmax, standardized Dmax; TMTV, total metabolic tumor volume.

**FIGURE 2:**
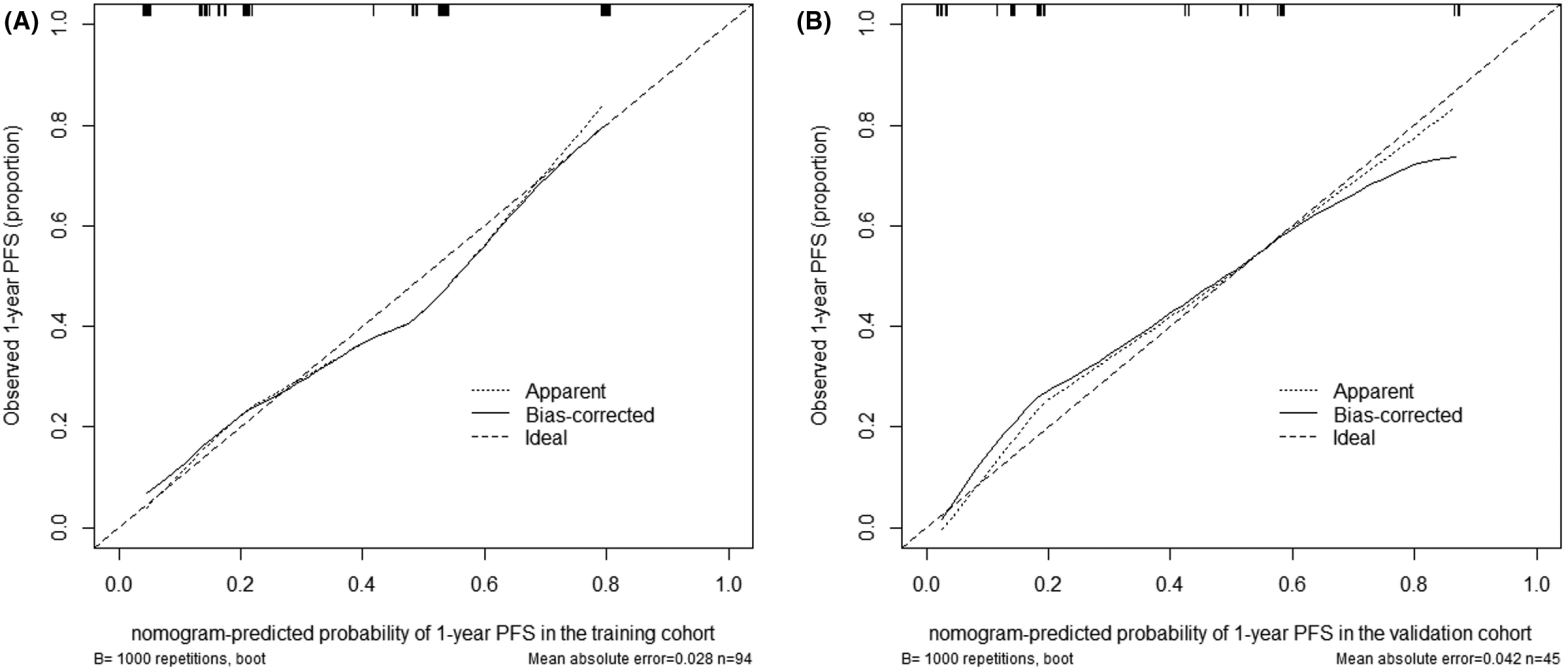
Calibration curve for predicting PFS at 1 year in the training (A) and the validation cohort (B). PFS is plotted on the *y*‐axis; prognostic model‐predicted probability of 1‐year PFS is plotted on the *x*‐axis. PFS, progression‐free survival.

**FIGURE 3:**
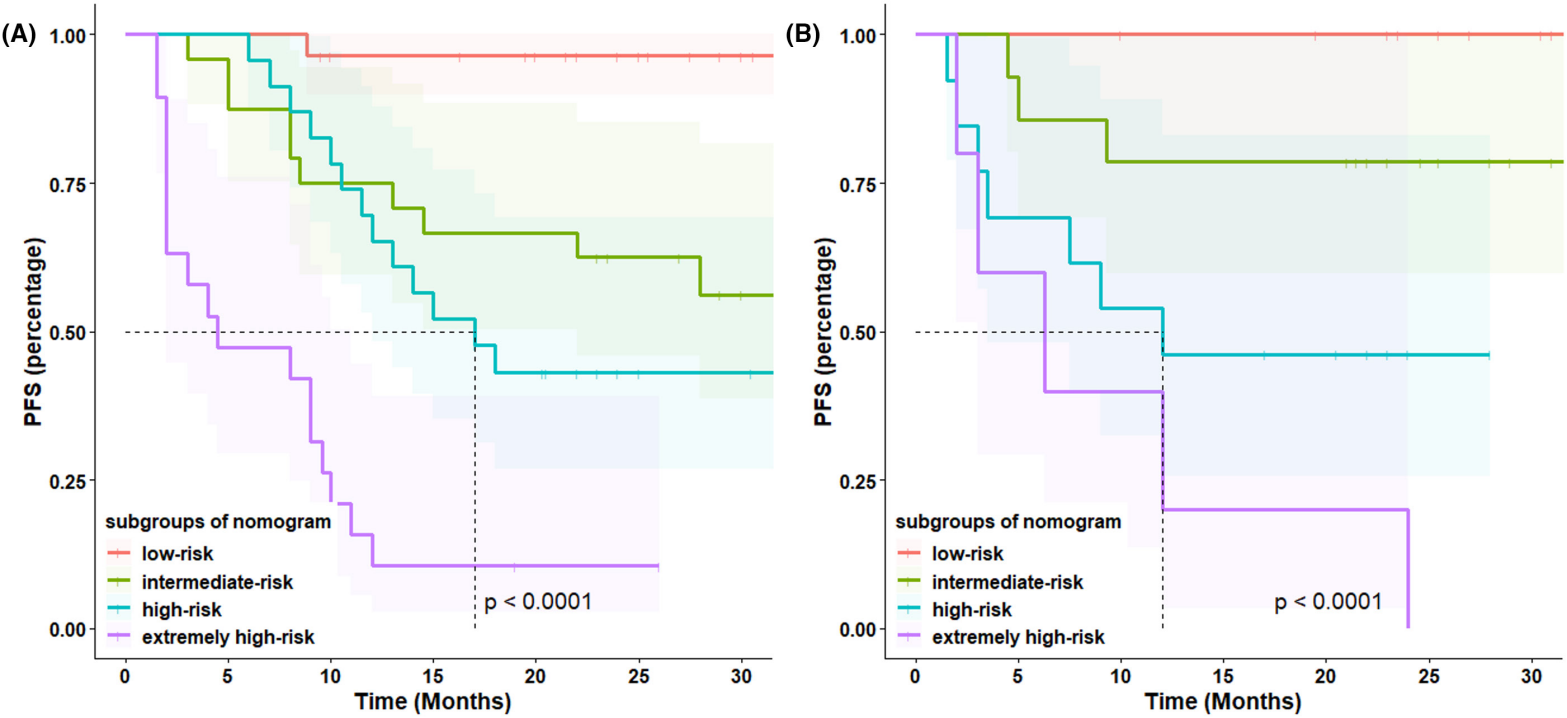
Nomogram to predict PFS in the training cohort (A) and in the validation cohort (B). Subgroups of patients with different nomogram scores (low‐risk: 0–72; intermediate‐risk: 83–100; high‐risk: 155–183 and extremely high‐risk: >183) showed distinct progression‐free survival (PFS) in the training (*n* = 94) and the validation cohort (*n* = 45).

Based on the result of multivariate Cox regression analysis, a nomogram was established to predict PFS of DLBCL patients (Figure 1). The 1‐year PFS prediction accuracy determined by the concordance index (C‐index) was 0.835 [95% CI: 0.753–0.917]. The calibration curves of the nomogram for 1‐year PFS prediction demonstrated promising agreement between the predicted and actual outcome in the training cohort (Figure 2A). Four risk groups with distinct PFS were identified in the training cohort based on quartile calculated by nomogram^1^: low‐risk (points: 0–72; 29.8%)^2^; intermediate‐risk (points: 83–100; 25.5%)^3^; high‐risk (points:155–183; 24.5%)^4^; extremely high‐risk (points: >183; 20.2%). In these four groups, 1‐year PFS was 96.4%, 75%, 65.2%, and 10.5% (*p* < 0.0001), respectively (Figure 3A).

In the validation cohort, the C‐index of 1‐year PFS prediction was 0.868 [95% CI: 0.765–0.970]. Similarly, good agreement between the predicted and actual 1‐year PFS of the nomogram was observed (Figure 2B). In total, 28.9% of the patients from the validation cohort were classified as low‐risk, 31.1% as intermediate‐risk, 28.9% as high‐risk, and 11.1% as extremely high‐risk group, with 1‐year PFS rate corresponding to 100%, 78.6%, 46.2%, and 20.0%(*p* < 0.0001), respectively (Figure 3B).

#### 3.3.1 Comparison of PFS between different models

**FIGURE 4:**
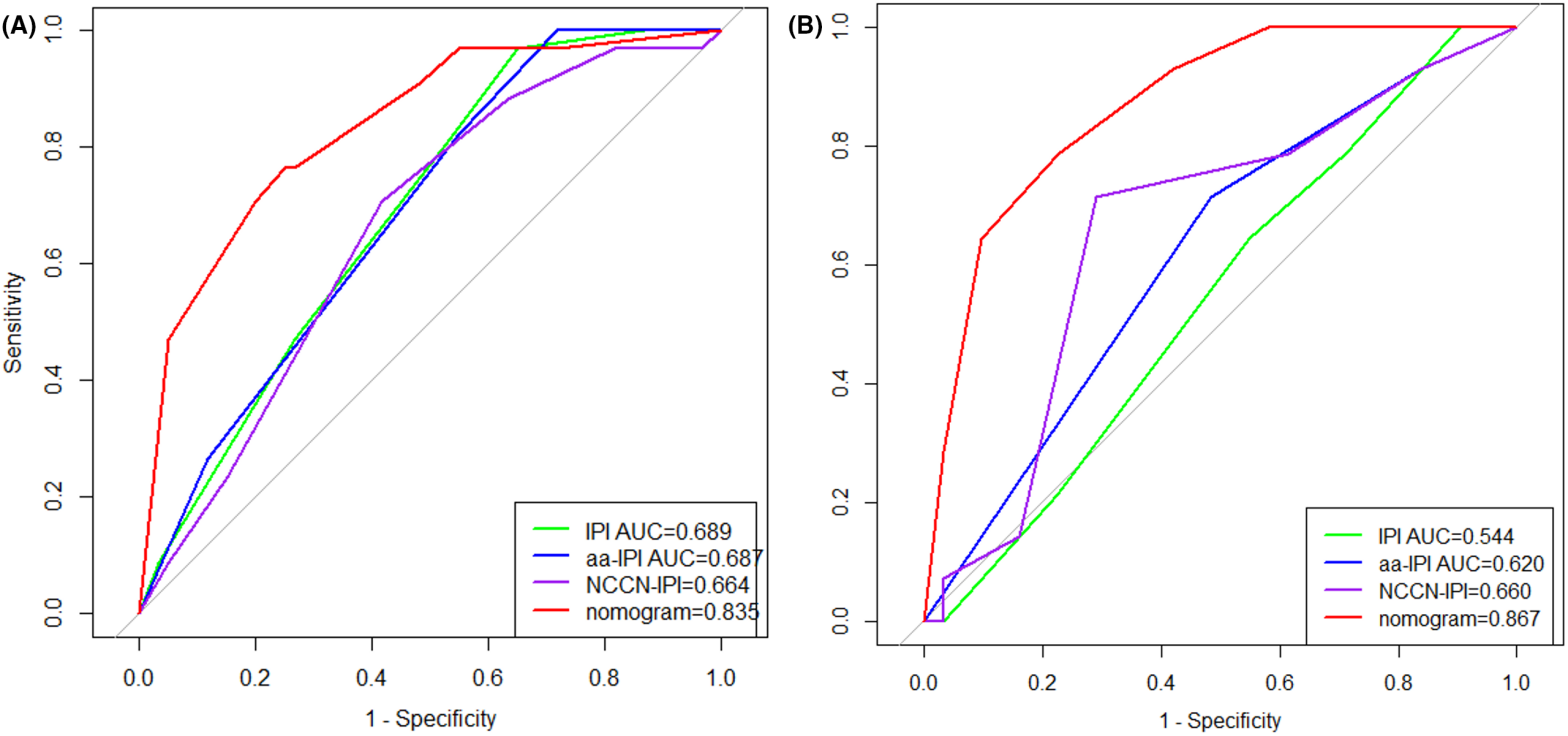
ROC curves and the AUCs at 1 year to assess the prediction performance of the prognostic model compared with IPI, aa‐IPI, and NCCN‐IPI in the training (A) and validation cohort (B). aa‐IPI, age‐adjusted‐IPI; AUCs, areas under the curve; IPI, the International Prognostic Index; NCCN‐IPI, National Comprehensive Cancer Network IPI; PFS, progression‐free survival; ROC, receiver operating characteristic.

The predictive power of the 1‐year PFS prediction of the nomogram was compared to the current IPI, aa‐IPI, and NCCN‐IPI risk stratification models. In the training cohort, ROC analysis showed that the nomogram had higher prognostic accuracy for 1‐year PFS than the IPI (AUC: 0.835 vs. 0.689, *p* = 0.007), aa‐IPI (AUC: 0.835 vs. 0.687, *p* = 0.007) and NCCN‐IPI (AUC: 0.835 vs. 0.664, *p* = 0.001) (Figure 4A). Similarly, upon external validation, ROC analysis also showed the nomogram model demonstrated higher prognostic accuracy for 1‐year PFS prediction than the IPI (AUC: 0.867 vs. 0.544, *p* < 0.001), aa‐IPI (AUC: 0.868 vs. 0.620, *p* = 0.011), and NCCN‐IPI risk scores (AUC: 0.868 vs. 0.660, *p* = 0.023) (Figure 4B).

## 4 DISCUSSION

Early identification of patients with DLBCL who are rapidly progressing under conventional therapy is needed to aid stratification for innovative treatment. Based on the data from the training cohort and validation cohort, we established and validated a nomogram that incorporated pretreatment TMTV, SDmax, and *TP53* status to predict PFS of DLBCL patients, showing higher predictive performance than the clinical risk scores.

TMTV, which represents metabolic tumor burden, is significantly related to PFS and OS in DLBCL.^24^, ^25^ Different studies have proposed various calculation methods and determined numerous cutoff values ranging from 200 cm^3^ to 300 cm^3^.^11^, ^12^, ^26^ In the current study, baseline TMTV was determined using a semi‐automatic method (41% maximum standardized uptake value threshold) for each patient. It has been revealed that patients with a high TMTV were more prone to early progression and high TMTV has been identified as an independent predictive factor for PFS. Dmax, as a 3D feature simple to calculate with an intuitive interpretation, has been demonstrated to identify poor prognosis in patients with DLBCL, Hodgkin's lymphoma, or other lymphomas.^27^, ^28^, ^29^ Although it was less affected by height according to the LNH073B study,^28^ we think that it is more reasonable to use body surface area to standardize Dmax, considering the consistency of comparison. The cutoff point of the SDmax between studies remains controversial. Our research shows that the cutoff value of SDmax is shorter than that reported in the REMARC study.^15^ The reason may be due to race‐related factors and the heterogeneity of the samples included, for the non‐germinal center B‐cell‐like (GCB) group accounted for 65% of our cohort whereas it accounted for 52% in the REMARC study. Thus, a multicenter large‐scale cohort study is still needed for verification.

The gene expression profile of DLBCL reflects heterogeneity and is of therapeutic importance.^16^, ^17^, ^30^ *TP53* mutations in DLBCL has been found in 20–30% of DLBCL patients^31^, ^32^ and is often associated with treatment resistance, especially mutations in the *TP53* DNA‐binding domains that cover exons 5–9.^33^, ^34^, ^35^ In this study *TP53* mutations were significantly associated with poor early prognosis. Our study did not detect the *TP53* deletion by fluorescence in situ hybridization, and we defined patients with >50% mutations as the A53‐like subtype. Univariate analysis showed the prognosis of patients in A53‐like subgroup was worse but considering the limitations of detection method and the limited number of patients, it was not included in multivariate analysis.

Several large multicenter studies have reported that MCD‐like type DLBCL cases associated with old age, extranodal involvement, and activated B‐cell‐like origin had a poor prognosis.^36^, ^37^ Our follow‐up showed no significant difference during MCD‐like subtype and non‐MCD‐like subtype. Of the 18 patients with the MCD‐like subtype, 15 received first‐line immunochemotherapy combined with a BTK inhibitor (BTKi). This confirms that standard immunochemotherapy combined with targeted therapy is worthy of further research to improve the prognosis of MCD‐like subtype.^38^, ^39^ No significant difference for CR and PFS was observed based on the gene mutations except *TP53* and therefore, longer follow‐up and larger cohort verification are still needed.

Compared with the existing clinical models, such as IPI, aa‐IPI, and NCCN‐IPI, the nomogram has a better predictive power for the rapid progression in each cohort. It is indicated the predictive model not only helps to predict prognosis but also contributes to future clinical trials design. It might be meaningful to explore novel therapies or intensive combined therapies with more effectiveness for these high‐risk cases based on the new model. It is also warranted to explore the reduction in the chemotherapy cycles and the adjustment of the interval of reexamination for the low‐risk population. In the future, prospective trials are needed to establish more individualized therapies as suitable treatment for patients classified into different risk groups based on the predictive model.

Although the nomogram showed good accuracy in predicting prognosis, it still had the following limitations. First, the cutoff points of TMTV and SDmax are still cohort‐dependent, being generated by ROC analysis of different measurement methods. The lack of agreement on the optimal cutoff points limited the use of PET parameters in routine clinical practice. Researchers have tried to use a segmentation method with a fixed threshold instead of the widely used percentage threshold at 41% of SUVmax to solve this problem.^40^ Currently, it is unknown how to best use the parameters of PET/CT. Second, this retrospective study included a high proportion of patients with advanced stage, with more than 70% of patients with III–IV stage, which may affect the distribution of *TP53* mutations. Finally, this retrospective study may lead to a certain degree of selection bias, thus the predictive ability of the nomogram should be further validated in larger and prospective studies.

## 5 CONCLUSIONS

In conclusion, the combination of baseline PET/CT metrics and *TP53* mutations detected by NGS is a strong predictor of short‐term prognosis in DLBCL. These results are worthy of further evaluation in other large cohorts and verification requires a longer follow‐up time. Increasing evidence shows that a next‐generation prognostic model may include PET scanning indicators and genetic factors to identify high‐risk patients early, guide clinical medication, and ultimately achieve individualized treatment.

## AUTHOR CONTRIBUTIONS

**Cong Liu:** Conceptualization (equal); data curation (equal); investigation (equal); methodology (equal); software (equal); visualization (equal); writing – original draft (lead). **Pengyue Shi:** Data curation (equal); investigation (equal); methodology (equal); software (equal); validation (equal). **Zhenjiang Li:** Data curation (equal); methodology (equal); software (equal); visualization (equal). **Baosheng Li:** Conceptualization (equal); supervision (equal); validation (equal); writing – review and editing (equal). **Zengjun Li:** Conceptualization (equal); project administration (equal); supervision (equal); validation (equal); writing – review and editing (equal).

## FUNDING INFORMATION

This work was supported in part by the National Natural Science Foundation of China (No. 82102173) and the 2021 Shandong Medical Association Clinical Research Fund: Qilu Special Project (No. YXH2022X02198).

## CONFLICT OF INTEREST STATEMENT

The authors declare that there is no conflict of interest.

## ETHICS STATEMENT

The study involving human participants was conducted under the approval and supervision of the Medical Ethical Committee of Shandong Cancer Hospital and Institute (No. SDTHEC2022007008). Written informed consent for participation was not required for this study in accordance with the national legislation and the institutional requirements. The waiver of informed consent was approved by the Medical Ethical Committee of Shandong Cancer Hospital and Institute. This study conformed to the provisions of the Declaration of Helsinki.

## Supporting information


Table S1.

Table S2.

Figure S1.

## Data Availability

The data that support the findings of this study are available from the corresponding author upon reasonable request.
