# Supplementary material for: A nomogram for predicting the rapid progression of diffuse large B‐cell lymphoma established by combining baseline PET/CT total metabolic tumor volume, lesion diffusion, and TP53 mutations
Source: Cancer Med. 2023 Jun 27;12(16):16734–43. doi: 10.1002/cam4.6295 (PMC10501242; doi:10.1002/cam4.6295)
Supplement: Supplementary file 1 — Table S1. [file CAM4-12-16734-s003.docx]

**Table S1 Gene mutation frequencies by the training (A) and validation (B) cohort.**

| **gene** | Training cohort  (n=94) | Validation cohort  (n =45) | Total  (n =139) | P value |
| --- | --- | --- | --- | --- |
| *TP53* | 35(37.23%) | 12(26.67%) | 47(33.81%) | 0.2536 |
| *ITPKB* | 5(5.32%) | 6(13.33%) | 11(7.91%) | 0.1752 |
| *JAK2* | 1(1.06%) | 0(0.00%) | 1(0.72%) | 1.0000 |
| *KMT2C* | 12(12.77%) | 4(8.89%) | 16(11.51%) | 0.5824 |
| *KMT2D* | 26(27.66%) | 9(20.00%) | 35(25.18%) | 0.4059 |
| *MEF2B* | 9(9.57%) | 3(6.67%) | 12(8.63%) | 0.7511 |
| *MFHAS1* | 8(8.51%) | 1(2.22%) | 9(6.47%) | 0.2711 |
| *MYC* | 10(10.64%) | 6(13.33%) | 16(11.51%) | 0.7771 |
| *MYD88* | 32(34.04%) | 14(31.11%) | 46(33.09%) | 0.8477 |
| *NOTCH1* | 8(8.51%) | 2(4.44%) | 10(7.19%) | 0.4996 |
| *NOTCH2* | 7(7.45%) | 3(6.67%) | 10(7.19%) | 1.0000 |
| *PAX5* | 5(5.32%) | 5(11.11%) | 10(7.19%) | 0.2927 |
| *PIM1* | 34(36.17%) | 17(37.78%) | 51(36.69%) | 0.8532 |
| *SGK1* | 6(6.38%) | 3(6.67%) | 9(6.47%) | 1.0000 |
| *SOCS1* | 9(9.57%) | 4(8.89%) | 13(9.35%) | 1.0000 |
| *STAT3* | 4(4.26%) | 3(6.67%) | 7(5.04%) | 0.6815 |
| *STAT6* | 8(8.51%) | 2(4.44%) | 10(7.19%) | 0.4996 |
| *TET2* | 10(10.64%) | 3(6.67%) | 13(9.35%) | 0.5476 |
| *TNFAIP3* | 16(17.02%) | 6(13.33%) | 22(15.83%) | 0.6299 |
| *TNFRSF14* | 7(7.45%) | 2(4.44%) | 9(6.47%) | 0.7180 |
| *XPO1* | 6(6.38%) | 2(4.44%) | 8(5.76%) | 1.0000 |
| *ARID1B* | 9(9.57%) | 5(11.11%) | 14(10.07%) | 0.7701 |
| *ATM* | 7(7.45%) | 1(2.22%) | 8(5.76%) | 0.4370 |
| *B2M* | 11(11.70%) | 10(22.22%) | 21(15.11%) | 0.1301 |
| *BCL10* | 5(5.32%) | 5(11.11%) | 10(7.19%) | 0.2927 |
| *BCL2A* | 5(5.32%) | 4(8.89%) | 9(6.47%) | 0.4707 |
| *BCL6A* | 3(3.19%) | 2(4.44%) | 5(3.60%) | 0.6587 |
| *BTG1* | 19(20.21%) | 8(17.78%) | 27(19.42%) | 0.8215 |
| *CARD11* | 17(18.09%) | 9(20.00%) | 26(18.71%) | 0.8183 |
| *CCND3* | 8(8.51%) | 8(17.78%) | 16(11.51%) | 0.1539 |
| *CD58* | 10(10.64%) | 6(13.33%) | 16(11.51%) | 0.7771 |
| *CD79A* | 5(5.32%) | 0(0.00%) | 5(3.60%) | 0.1744 |
| *CD79B* | 26(27.66%) | 9(20.00%) | 35(25.18%) | 0.4059 |
| *CDKN2A* | 6(6.38%) | 4(8.89%) | 10(7.19%) | 0.7273 |
| *PRDM1* | 12(12.77%) | 5(11.11%) | 17(12.23%) | 1.0000 |
| *CIITA* | 7(7.45%) | 2(4.44%) | 9(6.47%) | 0.7180 |
| *CREBBP* | 16(17.02%) | 5(11.11%) | 21(15.11%) | 0.4531 |
| *EP300* | 11(11.70%) | 8(17.78%) | 19(13.67%) | 0.4288 |
| *EPHA7* | 2(2.13%) | 2(4.44%) | 4(2.88%) | 0.5949 |
| *EZH2* | 5(5.32%) | 1(2.22%) | 6(4.32%) | 0.6638 |
| *FAS* | 7(7.45%) | 4(8.89%) | 11(7.91%) | 0.7471 |
| *GNA13* | 14(14.89%) | 3(6.67%) | 17(12.23%) | 0.2677 |
| *IRF8* | 4(4.26%) | 1(2.22%) | 5(3.60%) | 1.0000 |
