# Supplementary material for: A nomogram for predicting the rapid progression of diffuse large B‐cell lymphoma established by combining baseline PET/CT total metabolic tumor volume, lesion diffusion, and TP53 mutations
Source: Cancer Med. 2023 Jun 27;12(16):16734–43. doi: 10.1002/cam4.6295 (PMC10501242; doi:10.1002/cam4.6295)
Supplement: Supplementary file 2 — Table S2. [file CAM4-12-16734-s002.docx]

**Table S2 Univariate analysis for CR and PFS in 139 DLBCL patients of 43 genes and subtypes**

| Variable | CR | | | | |  | | PFS | | |
| --- | --- | --- | --- | --- | --- | --- | --- | --- | --- | --- |
|  | Odds ratio | | 95% confidence  interval | P value |  | | Hazard ratio | | 95% confidence  interval | P value |
| *ITPKB* | 0.256 | | 0.032–2.069 | 0.201 |  | | 2.885 | | 0.703–11.837 | 0.141 |
| *JAK2†* | - | | - | - |  | | - | | - | - |
| *KMT2C* | 2.411 | | 0.827–7.030 | 0.107 |  | | 0.540 | | 0.264-1.103 | 0.091 |
| *KMT2D* | 1.970 | | 0.864–4.488 | 0.107 |  | | 0.618 | | 0.353–1.084 | 0.093 |
| *MEF2B* | 1.424 | | 0.402–5.041 | 0.583 |  | | 0.579 | | 0.262–1.279 | 0.177 |
| *MFHAS1* | 0.326 | | 0.039-2.703 | 0.299 |  | | 1.385 | | 0.433-4.434 | 1.385 |
| *MYC* | 0.604 | | 0.162–2.253 | 0.453 |  | | 1.768 | | 0.639–4.889 | 0.272 |
| *MYD88L265R* | 0.423 | | 0.161–1.116 | 0.082 |  | | 1.530 | | 0.808–2.897 | 0.192 |
| *NOTCH1* | 1.939 | | 0.515-7.300 | 0.327 |  | | 0.494 | | 0.224-1.092 | 0.082 |
| *NOTCH2* | 1.197 | | 0.293-4.895 | 0.802 |  | | 0.963 | | 0.348-2.663 | 0.941 |
| *PAX5* | 1.197 | | 0.293-4.895 | 0.802 |  | | 1.390 | | 0.434-4.450 | 0.579 |
| *PIM1* | 0.379 | | 0.158--0.109 | 0.061 |  | | 1.604 | | 0.906-2.838 | 0.105 |
| *SGK1* | 0.504 | | 0.056-4.532 | 0.541 |  | | 0.935 | | 0.338-2.587 | 0.897 |
| *SOCS1* | 0.726 | | 0.141-3.747 | 0.702 |  | | 2.230 | | 0.696-7.140 | 0.177 |
| *STAT3‡* | - | | - | - |  | | 1.558 | | 0.380-6.393 | 0.538 |
| *STAT6* | 0.671 | | 0.136-3.317 | 0.625 |  | | 1.494 | | 0.467-4.783 | 0.499 |
| *TET2* | 0.473 | | 0.100-2.241 | 0.345 |  | | 1.453 | | 0.525-4.018 | 0.472 |
| *TNFAIP3* | 1.353 | | 0.504-3.637 | 0.549 |  | | 1.382 | | 0.626-3.052 | 0.423 |
| *TNFRSF14* | 3.828 | | 0.969-15.126 | 0.056 |  | | 0.721 | | 0.261-1.996 | 0.529 |
| *XPO1* | 1.712 | | 0.388-7.548 | 0.478 |  | | 0.693 | | 0.250-1.917 | 0.480 |
| *ARID1B* | 0.429 | | 0.091-2.013 | 0.283 |  | | 0.998 | | 0.428-2.330 | 0.997 |
| *ATM* | 3.939 | | 0.817-18.994 | 0.088 |  | | 0.587 | | 0.234-1.473 | 0.257 |
| *B2M* | 1.889 | | 0.712-5.009 | 0.201 |  | | 0.683 | | 0.344-1.354 | 0.274 |
| *BCL10* | 1.197 | | 0.293-4.895 | 0.802 |  | | 1.383 | | 0.432-4.426 | 0.585 |
| *BCL2A* | 1.412 | | 0.334-5.959 | 0.639 |  | | 1.169 | | 0.365-3.743 | 0.792 |
| *BCL6A* | 1.886 | | 0.302-11.758 | 0.497 |  | | 0.642 | | 0.200-2.054 | 0.455 |
| *BTG1* | 0.287 | | 0.081-1.017 | 0.053 |  | | 1.709 | | 0.808-3.614 | 0.161 |
| *CARD11* | 0.603 | | 0.209-1.736 | 0.348 |  | | 1.575 | | 0.745-3.331 | 0.235 |
| *CCND3* | 1.293 | | 0.417-4.007 | 0.657 |  | | 1.171 | | 0.530-2.585 | 0.696 |
| *CD58* | 0.909 | | 0.274-3.018 | 0.876 |  | | 1.213 | | 0.520-2.830 | 0.655 |
| *CD79A* | 1.886 | | 0.302-11.758 | 0.497 |  | | 0.730 | | 0.178-2.996 | 0.662 |
| *CD79B* | 0.255 | | 0.069-1.004 | 0.051 |  | | 1.521 | | 0.786-2.942 | 0.213 |
| *CDKN2A* | 0.287 | | 0.035-2.348 | 0.244 |  | | 1.073 | | 0.388-2.967 | 0.892 |
| *PRDM1* | 0.331 | | 0.072-1.526 | 0.156 |  | | 1.734 | | 0.692-4.347 | 0.240 |
| *CIITA* | 2.352 | | 0.596-9.280 | 0.222 |  | | 0.838 | | 0.303-2.318 | 0.734 |
| *CREBBP* | 0.840 | | 0.284-2.481 | 0.752 |  | | 1.059 | | 0.501-2.240 | 0.880 |
| *EP300* | 0.982 | | 0.327-2.946 | 0.974 |  | | 0.969 | | 0.458-2.048 | 0.934 |
| *EPHA7* | 0.917 | | 0.092-9.098 | 0.941 |  | | 1.343 | | 0.186-9.716 | 0.771 |
| *EZH2* | 2.912 | | 0.561-15.117 | 0.203 |  | | 0.647 | | 0.202-2.074 | 0.464 |
| *FAS* | 0.590 | | 0.122-2.869 | 0.514 |  | | 1.651 | | 0.516-5.286 | 0.398 |
| *GNA13* | 2.147 | | 0.751-6.135 | 0.154 |  | | 0.789 | | 0.373-1.669 | 0.536 |
| *IRF8* | 4.412 | | 0.707-27.530 | 0.112 |  | | 0.441 | | 0.159-1.223 | 0.116 |
| *TP53* | 5.339 | | 2.383-11.962 | <0.001 |  | | 0.365 | | 0.215-0.620 | <0.001 |
| MCD-like | 0.307 | | 0.067-1.407 | 0.128 |  | | 1.674 | | 0.668-4.197 | 0.272 |
| A53-like | 4.414 | | 1.416-13.76 | 0.01 |  | | 0.319 | | 0.160-0.637 | 0.01 |
| N1-like | 0.681 | | 0.074-6.293 | 0.735 |  | | 0.590 | | 0.184-1.892 | 0.375 |
| BN2-like | | 0.539 | 0.061-4.771 | 0.578 |  | | 2.845 | | 0.393-20.576 | 0.300 |
| EZH2-like | | 1.389 | 0.122-15.784 | 0.791 |  | | 1.273 | | 0.176-9.205 | 0.811 |
| ST2-like | | 0.287 | 0.035-2.348 | 0.244 |  | | 2.345 | | 0.571-9.623 | 0.237 |

† Only one patient had JAK2 mutation

‡ Seven patients with STAT3 mutations all get CR.
