# Supplementary figures and images for: A nomogram for predicting the rapid progression of diffuse large B‐cell lymphoma established by combining baseline PET/CT total metabolic tumor volume, lesion diffusion, and TP53 mutations
Source: Cancer Med. 2023 Jun 27;12(16):16734–43. doi: 10.1002/cam4.6295 (PMC10501242; doi:10.1002/cam4.6295)

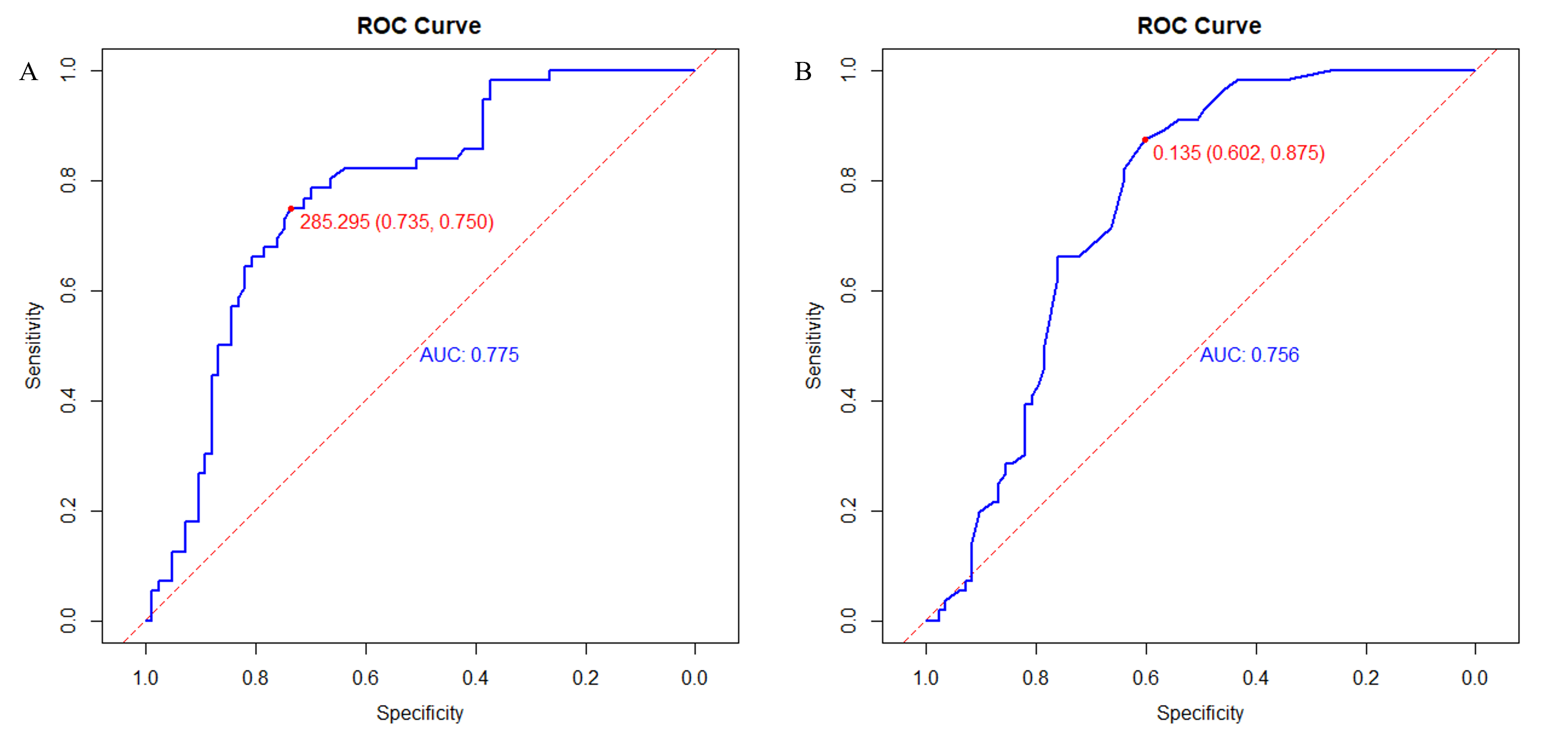

Supplement: Supplementary file 3 — Figure S1. [file CAM4-12-16734-s001.tif]
